# A U-shaped association between selenium intake and cancer risk

**DOI:** 10.1038/s41598-024-66553-5

**Published:** 2024-09-13

**Authors:** Ngoan Tran Le, Yen Thi-Hai Pham, Chung Thi-Kim Le, Linh Thuy Le, Thanh-Do Le, Hang Viet Dao, Toan H. Ha, Suresh V. Kuchipudi, Hung N. Luu

**Affiliations:** 1 Institute of Research and Development, Duy Tan University, Da Nang, Vietnam; 2 Department of Occupational Health, Institute of Preventive Medicine and Public Health, Hanoi Medical University, 1 Ton That Tung, Hanoi, Vietnam; 3 The University of Pittsburgh Medical Center Hillman Cancer Center, Pittsburgh, PA USA; 4 Department of Epidemiology, School of Public Health, University of Pittsburgh, Pittsburgh, PA USA; 5 Laboratory Center, School of Preventive Medicine and Public Health, Hanoi Medical University, Hanoi, Vietnam; 6 Laboratory of Embryology and Genetics of Human Malformation, Imagine Institute, INSERM UMR, Paris, France; 7 Institute for Global Health Innovations, Duy Tan University, Da Nang, Vietnam; 8 Department of Internal Medicine, Hanoi Medical University, Hanoi, Vietnam; 9 Department of Infectious Disease and Microbiology, School of Public Health, University of Pittsburgh, Pittsburgh, PA USA

**Keywords:** Selenium intake, Dietary, Cancer risk, Case–control study, Vietnam, Cancer, Chemical biology, Biomarkers, Diseases, Risk factors

## Abstract

While selenium is a cofactor of several antioxidant enzymes against cancer and is essential for human health, its excess intake may also be harmful. Though a safe intake of selenium has recently been recommended, it is not well understood in the Asian population. We aimed to determine the association between dietary intake of selenium and cancer risk in a case–control study of 3758 incident cancer cases (i.e., stomach, colon, rectum, lung cancers, and other sites) and 2929 control subjects in Vietnam. Daily intake of selenium was derived from a semiquantitative food frequency questionnaire. The unconditional logistic regression model was used to calculate the odds ratios (ORs) and 95% confidence intervals (CIs) for the association between selenium intake and cancer risk. We observed a U-shaped association between selenium intake and cancer risk. A safe intake ranged from 110.8 to 124.4 µg/day (mean 117.8 µg/day). Compared to individuals with the safe intake of selenium, individuals with the lowest intake (i.e., 27.8–77.2 µg/day) were associated with an increased risk of cancer (OR = 3.78, 95% CI 2.89–4.95) and those with the highest intake (169.1–331.7 µg/day) also had an increased cancer risk (OR = 1.86, 95% CI 1.45–2.39). A U-shaped pattern of association between selenium intake and cancer risk was stronger among participants with body mass index (BMI) < 23 kg/m^2^ and never smokers than BMI ≥ 23 kg/m^2^ and ever smokers (*P’s*_*heterogeneity*_ = 0.003 and 0.021, respectively) but found in both never and ever-drinkers of alcohol (*P*_heterogeneity_ = 0.001). A U-shaped association between selenium intake and cancer risk was seen in cancer sites of the stomach, colon, rectum, and lung cancers. In summary, we found a U-shaped association between selenium intake and cancer risk and a safe selenium intake (mean: 117.8 µg/day) in the Vietnamese population. Further mechanistic investigation is warranted to understand better a U-shaped association between selenium intake and cancer risk.

## Introduction

Selenium is an essential element of selenoproteins, playing an important role in different biological functions, including DNA synthesis, antioxidant defense, fertility and reproduction, and thyroid hormone formation^1^. Selenium species can be classified into selenium-containing organic compounds (e.g., selenomethionine, selenocysteine) and inorganic forms (selenate, selenite)^2^. Tissue selenium supply is dependent on the plasma selenium transporter selenoprotein P^3^. Prior animal model studies showed that loss of motor coordination and viability reduction of whole-body selenoprotein P knockout mice were exhibited 15 days after weaning^4^. The survival rate and neurological and reproductive impairments are circumvented by supplementing diets with an established nutritional requirement of 0.1 mg/kg selenium^4,5^.

Food consumption is the main source of selenium exposure, however, selenium exposure in humans can occur through different other routes, including drinking water, air or dietary supplements^6–8^. While selenium is essential for human health, it is also toxic^2,9^. It is critical to note that the range between an essential level and a toxic level of selenium is narrow, and the range of safe intake for selenium is still not well-defined^10,11^. Selenium toxicity results in hair and nail loss, blindness, or changes in the nervous system and skin lesions^12^. Acute selenium poisoning was recently described in the United States from ingestion of liquid dietary supplements that contained 200 times higher selenium content than labeled^13^. Toxic signs of selenium excess were observed in subjects with blood selenium levels of 13.3 μmol/L and above. In humans, an intake of up to 500 μg/day can be a tolerable level; yet other studies indicate that the maximum tolerable selenium level in the lumen could be from 1000 to 1500 μg/day^14^.

The recommended daily allowance of selenium intake differs between regulatory agencies. For example, the Recommended Dietary Allowance (RDA) for selenium is 55 μg/day for adults^1,15^. The US Institute of Medicine has set the tolerable upper intake level to 400 μg/day for adults^16^. The World Health Organization recommends 25 to 34 µg/day, depending on age and sex^17^. Vinceti et al. questioned the current upper limit of ‘safe intake’ and proposed a far lower ‘safe level’ for long-term usage (i.e., 20 μg/day for organic selenium) and a differentiation between organic and inorganic selenium sources^18^. While acute and chronic toxicity of high selenium exposure has been well-studied, the long-term effects (i.e., harmful) of low-dose selenium intake are not fully understood^19^. Also, because there may be differences in the biological activities of selenium in organic and inorganic forms^2,20^. Recently, the European Commission, the EFSA Panel on Nutrition, Novel Foods and Food Allergens (NDA) conducted a systematic review and identified the lowest‐observed‐adverse‐effect‐level (LOAEL) of 330 μg/day, a result from the Selenium and Vitamin E Cancer Prevention Trial (SELECT), a large randomized controlled trial in human^21^. Since this trial focused on the Caucasian population, there is still an urgently unmet need to understand better the safe intake of selenium among the Asian population, other than the Chinese^22,23^.

There has been inconclusive evidence about the role of selenium in cancer prevention. A recent review found that selenium was associated with reduced risk of stomach, colorectal, lung, breast, and bladder^24^. However, they also pointed out that these studies had significant limitations. No dose–response relation between selenium and cancer risk was observed. More importantly, the same review also showed that data from randomized clinical trials did not support the protective effect of selenium administration against cancers, including colorectal, non‐melanoma, skin, lung, breast, bladder, and prostate cancer^24^. We aimed to determine the association between dietary intake of selenium, both low- and high-levels, using a safe intake as a reference, and cancer risk in a large case–control study comprising 3758 cancer incidents and 2929 control patients in Vietnam.

## Methods

### Study population

We used data from a hospital-based case–control study in Vietnam that recruited participants from four leading facility hospitals in Hanoi between 2003 and 2019, breaking into four periods: (1) 2003–2006 period (n = 625 participants), (2) 2006–2007 period (n = 1342 participants), (3) 2008 period (n = 407 participants) and (4) 2018–2019 period (n = 4902 participants). Patients were newly diagnosed by histopathology and underwent surgery for cancer treatment at the Bach Mai Hospital, Viet Duc University Hospital, National Cancer Hospital, and Hanoi Medical University Hospital, four tertiary hospitals in Hanoi, Vietnam. Our study was performed in accordance with the relevant guidelines and regulations by two Institution Review Boards, including the Institution Review Board (IRB) approved the study protocol for Ethics in Biomedical Research—Hanoi Medical University (Approval number 3918/HMUIRB and the ethics committees of the IRB-International University of Health and Welfare, Japan (Approval number 19-Ig-17). All participants provided written informed consent. Detailed methods and initial results of this case–control study were published elsewhere^25–28^.

#### Cases recruitment

New cancer cases were recruited from four tertiary hospitals in Hanoi, Vietnam, including the Bach Mai University Hospital, Viet Duc University Hospital, National Cancer Hospital, and Hanoi Medical University Hospital. The recruited cancer sites included stomach (ICD-10: C16, n = 1,182), colon cancer (ICD-10: C18, n = 567), rectal cancer (ICD-10: C20, n = 482), lung (ICD-10: C34, n = 225), breast cancer (ICD-10: C50, n = 287), cervical cancer (ICD-10: C53, n = 45), ovary cancer (ICD-10: C56, n = 50) esophageal cancer (ICD-10: C15, n = 195), liver (ICD-10: C22, n = 68), nasopharynx (ICD-10: C11, n = 119), pancreas (ICD-10: C25, n = 37), bladder (ICD-10: C67, n = 40), thyroid (ICD-10: C73, n = 87), and other 26 cancer sties (n = 374). Consequently, a total of 3,758 new cancer patients were recruited in the current study.

The inclusion criteria were (1) those indicated for surgery when physically healthy enough to endure the surgery; (2) could communicate well by providing exposure data; (3) confirmed histopathology with cancer; and (4) who agreed to participate in the study. Exclusion criteria were (1) those who refused to participate in the study, (2) those who could not communicate in providing exposure data, and (3) those who had a change in diet due to illness. The recruitment process of cases has been published elsewhere^27–29^.

#### Controls recruitment

Controls were randomly chosen and matched with cases based on sex- and age (± 5 years). Control subjects were commonly diagnosed with prostate fibroma (n = 91), urinary tract stone (n = 704), biliary tract stone (n = 230), injury, or non-cancer surgery (n = 1970), making a total of 2995 control participants. Inclusion criteria were (1) those who did not have cancer at the time or in the past and (2) those who had good communication in providing exposure data and agreed to participate in the study. Exclusion criteria were (1) those who refused to participate in the study and (2) those who changed their diet due to health conditions or illness. The recruitment process of control was published elsewhere^27–29^. Due to (i) advanced matching of sex and age to get comparability between cases and controls, and (ii) more cancer patients were admitted to these four hospitals than non-cancer patients, by the end of the projects, we successfully recruited 3758 cancer patients and only 2995 control patients.

#### Exposure data collection

Using structured and validated questionnaires and face-to-face interviews, trained interviewers performed the interview at the outpatient departments or in-patient beds the day before the surgery. The exposure data of the cases and controls provided were validated and confirmed by the family members who prepared the foods. Information collected in the questionnaire included socio-demographics, body weight and height, lifetime tobacco use, occupational exposure, medical history, family history of cancer, and diet (details below in the Dietary Assessment). Additional information, including test results for HBV, HCV, HIV, and *H. pylori* (if any), was extracted from the medical records after the interview by the investigators.

### Dietary assessment

A semi-quantitative food frequency questionnaire (FFQ) was used to collect the average frequency of food intakes and portion sizes during the past 12 months for 85 food items commonly consumed by Vietnamese, based on the results of the dietary survey in general populations. The FFQ was first designed in 2003 and updated in 2017 based on the results of a survey asking to record food consumption in the past 24 h for three consecutive days in 158 households (n = 741 individuals) in 2003 and 298 households (n = 1327 individuals) in 2017. Food selection for the FFQ from a household survey made up to 90% of essential nutrients converted from the natural organic whole food used by the participating households^26,30,31^.

In the FFQ, study participants were asked how frequently they consumed the food and food group in 6 categories, ranging from *“6–11 times/year”, “1–3 times/month”, “1–2 times/week”, “3–4 times/week”, “5–6 times/week”*, and *“1–3 times/day”*, followed by a question on the amount of food consumed from three portion sizes (i.e., small, medium and large). The average daily intake of 95 nutrients and non-nutrient compounds, including selenium, was calculated for each participant using the Vietnamese Food Composition Database^32^. The FFQ was validated using two 24-h dietary recalls (24-HDRs) from October through December 2017, once every weekday and once every three consecutive other days, among 298 families (n = 1327 individuals). The Pearson correlation coefficients (*R*^2^) between the FFQ and 24-HDR ranged from 0.34 (for selenium) to 0.53 (for energy intake)^30,33^.

### Assessment of other covariates

Other covariate information was collected using a structured questionnaire. Such information, which was also included in the multivariable logistic regression models, were age (i.e., 15–29, 30–39, 40–49, 50–59, 60–69, ≥ 70 years of age), sex (male vs. female), the highest education level (i.e., primary school, secondary school, high school or higher), BMI (kg/m^2^, including < 18.5, 18.5–< 23, 23–24.9, ≥ 25), alcohol consumption (i.e., never vs. ever), family history of cancer (i.e., yes vs. no), smoking status (i.e., ever vs. never), history of diabetes (i.e., yes vs. no), coffee drinking (i.e., yes vs. no), total energy intake (kcal/day, tertile), and four periods of data collection (i.e., 2003–2006, 2006–2007, 2008, and 2018–2019).

### Statistical analysis

For continuous variables, means and standard deviations (SDs) were calculated, and for categorical variables, we computed counts and proportions. To compare the difference in distributions of continuous variables and categorical variables between cancer cases vs. control participants and across different levels of selenium intakes, we calculated the t-test and *χ*^2^ test, respectively.

We performed an unconditional logistic regression model to determine the association between selenium intake and risk of cancer by calculating odds ratios (ORs) and their 95% confidence intervals (CIs) across different levels of dietary selenium intake compared with the reference group. Since there is no preconceived notification of the recommended or safe selenium intake for Vietnamese, mean selenium intake at 117.8 µg/day (range: 110.8–124.4 µg/day) was used as a reference group in the current analysis. Since the second and third quartiles were close to each other in the lower range than the mean level, we decided to collapse them into one category. Finally, there were eight categories of selenium intake used in our analysis, including 67.4 µg/day (range: 27.8–77.2 µg/day), 86.8 µg/day (range: 77.3–95.0 µg/day), and 103.1 µg/day (range: 95.1–110.7 µg/day) as lower intakes of selenium and 130.9 µg/day (124.5–137 µg/day), 143.8 µg/day (137.1–151 µg/day), 159.2 µg/day (151.1–169.0 µg/day), and 186.3 µg/day (169.1–331.7 µg/day) as higher intakes of selenium. To see a U-shape association between selenium intake and cancer risk, we used point estimates (ORs) and their respective CIs, which were not dependent on the *P-value* and were shown to have different limitations as reported prior^34–37^. We estimated *P* for trend (*P*_trend_) for the estimated quantiles below a reference group and the estimated quantiles above a reference group.

We further conducted stratified analysis by sex, BMI (< 23 kg/m^2^ vs. ≥ 23 kg/m^2^), smoking status (ever vs. never), and alcohol drinking status (ever vs. never). The linear trend for cancer risk with selenium intake was tested based on the ordinal values of the lower and upper of the mean intake. The interactions between selected factors, including sex, BMI kg/m^2^, smoking status, alcohol drinking status, and selenium intake, were determined by including product terms between selenium intake and these factors in the multivariable logistic regression models. For specific cancer sites, we analyzed for cancer of the stomach (ICD-10: C16, n = 1,182), colon cancer (ICD-10: C18, n = 567), rectal cancer (ICD-10: C20, n = 482), and lung (ICD-10: C34, n = 225). All other cancer sites were grouped into “other cancers.” All statistical analysis used the Stata statistical package (Version 14.0—StataCorp LP., College Station, TX). We used 0.05 as a level of statistical significance in the current analysis.

### Ethics approval and consent to participate

We confirm that all methods were performed in accordance with the relevant guidelines and regulations by two Institution Review Boards, including the Institution Review Board (IRB) approved the study protocol for Ethics in Biomedical Research—Hanoi Medical University (Approval number 3918/HMUIRB dated 25 December 2018 and by the ethics committees of the IRB-International University of Health and Welfare, Japan (Approval number 19-Ig-17) dated 27 May 2019. All participants provided written informed consent. Study participants completed a questionnaire survey that allowed investigators access to data from routine laboratory tests of *H. pylori* infection and hepatitis viral infection.

Participants were anonymous, and personal information was coded, which cannot identify participants. Each participant was coded with an ID. Data is saved into a USB stick memory with a password.

## Results

Compared to cancer patients, controls were younger, had higher levels of education, higher BMI kg/m^2^, were less likely to be smokers or drinkers but more likely to be coffee drinkers, less likely to have a history of cancer, higher proportion of history of type 2 diabetes, more likely to be with blood groups AB and O, had lower total energy intake and higher levels of selenium intake (All *P’s* < 0.05). (Table 1). Compared to participants in the lowest intake of selenium (67.4 µg/day), participants in the highest level of selenium intake (188.3 µg/day) were younger, more likely to be male, had higher levels of education, more likely to have a history of cancer, lower BMI kg/m^2^, more likely to be smoker and coffee drinker but less likely to be alcohol drinker, had a higher proportion of history of type 2 diabetes, more likely to have blood groups of B and O, and had higher level of total energy intake (All *P’s* < 0.05) (Table 2).

**Table 1:** Distribution of selected characteristics among study participants.

Characteristics	Controls (n = 2995)	Cancer cases (n = 3758)	*P-value*
Age, (mean ± SD)	54.5 (± 12.2)	56.5 (± 11.9)	< 0.001
15–29	80 (2.7)	75 (2.0)	< 0.001
30–39	287 (9.6)	262 (7.0)	
40–49	622 (20.8)	613 (16.3)	
50–59	916 (30.6)	1189 (31.6)	
60–69	781 (26.1)	1142 (30.4)	
≥ 70	309 (10.3)	477 (12.7)	
Sex			
Men	1757 (58.7)	2267 (60.3)	0.167
Women	1238 (41.3)	1491 (39.7)	
Highest level of education			
Primary school	442 (14.8)	705 (18.8)	< 0.001
Secondary school	1322 (44.1)	1626 (43.3)	
High school or higher	1231 (41.1)	1427 (38.0)	
History of cancer			
No	2764 (92.3)	3416 (90.9)	0.042
Yes	231 (7.7)	342 (9.1)	
BMI, (mean ± SD)	21.3 (± 3.0)	20.1 (± 2.8)	< 0.001
< 18.5	557 (18.6)	1348 (35.9)	< 0.001
18.5–22.9	1630 (54.4)	1858 (49.4)	
23.0–24.9	509 (17.0)	391 (10.4)	
≥ 25	299 (10.0)	161 (4.3)	
Smoking status			
Never smoker	1822 (60.8)	2190 (58.3)	0.033
Ever smoker	1173 (39.2)	1568 (41.7)	
Alcohol consumption			
Never drinker	1706 (57.0)	1969 (52.4)	< 0.001
Ever drinker	1289 (43.0)	1789 (47.6)	
Coffee drinking status			
Never drinker	2289 (76.4)	3110 (82.8)	< 0.001
Ever drinker	706 (23.6)	648 (17.2)	
History of diabetes			
Yes	524 (17.5)	447 (11.9)	< 0.001
No	2471 (82.5)	3311 (88.1)	
Blood group^a^			
A	477 (20.8)	838 (30.0)	< 0.001
B	111 (4.8)	172 (6.2)	
AB	671 (29.2)	758 (27.1)	
O	1037 (45.2)	1027 (36.7)	
Total energy intake (kcal/day, mean ± SD)	1703.6 (± 441.4)	1485.5 (± 506.8)	< 0.001
Tertile 1	595 (19.9)	1664 (44.3)	
Tertile 2	1172 (39.1)	1076 (28.6)	
Tertile 3	1228 (41.0)	1018 (27.1)	< 0.001
Selenium intake (µg/day) (mean ± SD)	129 (± 34)	113.6 (± 38.6)	< 0.001
67.4 (27.8–77.2) µg/day	152 (5.1)	599 (15.9)	< 0.001
86.8 (77.3–95.0) µg/day	437 (14.6)	1063 (28.3)	
103.1 (95.1–110.7) µg/day	345 (11.5)	409 (10.9)	
117.8 (110.8–124.4) µg/day	424 (14.2)	324 (8.6)	
130.9 (124.5–137.0) µg/day	435 (14.5)	314 (8.4)	
143.8 (137.1–151.0) µg/day	419 (14.0)	332 (8.8)	
159.2 (151.1–169.1) µg/day	405 (13.5)	345 (9.2)	
188.3 (169.2–331.7) µg/day	378 (12.6)	372 (9.9)	

^a^Based on available data, SD is standard deviation, BMI is body Mass Index (Asian category).

**Table 2:** Distribution of selected characteristics among study participants according to mean levels of selenium intake.

Selenium intake, µg/day, mean	67.4 µg/day	86.8 µg/day	103.1 µg/day	117.8 µg/day	130.9 µg/day	143.8 µg/day	159.2 µg/day	188.3 µg/day	*P-value*
Range (min–max), µg/day	27.8–77.2 µg/day	77.3–95.0 µg/day	95.1–110.7 µg/day	110.8–124.4 µg/day	124.5–137.0 µg/day	137.1–151.0 µg/day	151.1–169.0 µg/day	169.1–331.7 µg/day	
Control, n (%) ^a^	152 (20.2)	437 (29.1)	345 (45.8)	424 (56.7)	435 (58.1)	419 (55.8)	405 (54.0)	378 (50.4)	< 0.001
Cancer, n (%)	599 (79.8)	1,063 (70.9)	409 (54.2)	324 (43.3)	314 (41.9)	332 (44.2)	345 (46.0)	372 (49.6)	
Age, mean(± SD)	57 (± 12)	56.1 (± 12.1)	56.6 (± 12.3)	57.4 (± 12.5)	56.4 (± 12)	55 (± 12)	53.9 (± 11)	52 (± 11.9)	
15–29, n (%)	10 (1.3)	35 (2.3)	14 (1.9)	18 (2.4)	14 (1.9)	21 (2.8)	17 (2.3)	26 (3.5)	< 0.001
30–39, n (%)	46 (6.1)	128 (8.5)	68 (9.9)	53 (7.1)	62 (8.3)	57 (7.6)	58 (7.7)	77 (10.3)	
40–49, n (%)	126 (16.8)	236 (15.7)	111 (14.7)	110 (14.7)	122 (16.3)	154 (20.5)	172 (22.9)	204 (27.2)	
50–59, n (%)	250 (33.3)	454 (30.3)	233 (30.9)	207 (27.7)	208 (27.8)	243 (32.4)	268 (35.7)	242 (32.3)	
60–69, n (%)	213 (28.4)	467 (31.1)	228 (30.2)	241 (32.2)	246 (32.8)	190 (25.3)	185 (24.7)	153 (20.4)	
≥ 70, n (%)	106 (14.1)	180 (12.0)	100 (13.3)	119 (15.9)	97 (13.0)	86 (11.5)	50 (6.7)	48 (6.4)	
Sex									
Men, n (%)	392 (52.2)	830 (55.3)	417 (55.3)	417 (55.7)	443 (59.1)	459 (61.1)	518 (69.1)	548 (73.1)	< 0.001
Women, n (%)	359 (47.8)	670 (44.7)	337 (44.7)	331 (44.3)	306 (40.9)	292 (38.9)	232 (30.9)	202 (26.9)	
Highest level of education									
Primary school, n (%)	209 (27.8)	211 (14.1)	138 (18.3)	127 (17.0)	113 (15.1)	119 (15.8)	106 (14.1)	124 (16.5)	< 0.001
Secondary school, n (%)	322 (42.9)	605 (40.3)	327 (43.4)	326 (43.6)	337 (45.0)	326 (43.4)	348 (46.4)	357 (47.6)	
High school or higher, n (%)	220 (29.3)	684 (45.6)	289 (38.3)	295 (39.4)	299 (39.9)	306 (40.7)	296 (39.5)	269 (35.9)	
History of cancer									
No, n (%)	693 (92.3)	1,407 (93.8)	681 (90.3)	685 (91.6)	681 (90.9)	678 (90.3)	675 (90.0)	680 (90.7)	0.019
Yes, n (%)	58 (7.7)	93 (6.2)	73 (9.7)	63 (8.4)	68 (9.1)	73 (9.7)	75 (10.0)	70 (9.3)	
BMI, mean (SD)	21.1 (± 2.8)	20.6 (± 2.8)	21.0 (± 3.1)	20.9 (± 2.9)	20.5 (± 3.1)	20.4 (± 3.0)	20.6 (± 2.9)	20.5 (± 3.0)	
< 18.5, n (%)	198 (26.4)	500 (33.3)	187 (24.8)	174 (23.3)	213 (28.4)	218 (29.0)	206 (27.5)	209 (27.9)	< 0.001
18.5–22.9, n (%)	388 (51.7)	760 (50.7)	379 (50.3)	411 (54.9)	365 (48.7)	401 (53.4)	393 (52.4)	391 (52.1)	
23.0–24.9, n (%)	116 (15.4)	150 (10.0)	118 (15.6)	112 (15.0)	125 (16.7)	81 (10.8)	96 (12.8)	102 (13.6)	
≥ 25, n (%)	49 (6.5)	90 (6.0)	70 (9.3)	51 (6.8)	46 (6.1)	51 (6.8)	55 (7.3)	48 (6.4)	
Smoking status									
Never smoker, n (%)	470 (62.6)	955 (63.7)	457 (60.6)	451 (60.3)	452 (60.3)	448 (59.7)	388 (51.7)	391 (52.1)	< 0.001
Ever smoker, n (%)	281 (37.4)	545 (36.3)	297 (39.4)	297 (39.7)	297 (39.7)	303 (40.3)	362 (48.3)	359 (47.9)	
Alcohol consumption									
Never drinker, n (%)	342 (45.5)	843 (56.2)	458 (60.7)	440 (58.8)	428 (57.1)	407 (54.2)	364 (48.5)	393 (52.4)	< 0.001
Ever drinker, n (%)	409 (54.5)	657 (43.8)	296 (39.3)	308 (41.2)	321 (42.9)	344 (45.8)	386 (51.5)	357 (47.6)	
Coffee drinking status									
Never drinker, n (%)	649 (86.4)	1,279 (85.3)	632 (83.8)	609 (81.4)	578 (77.2)	571 (76.0)	535 (71.3)	546 (72.8)	< 0.001
Ever drinker, n (%)	102 (13.6)	221 (14.7)	122 (16.2)	139 (18.6)	171 (22.8)	180 (24.0)	215 (28.7)	204 (27.2)	
History of diabetes									
Yes, n (%)	69 (9.2)	103 (6.9)	55 (7.3)	79 (10.6)	137 (18.3)	152 (20.2)	180 (24.0)	196 (26.1)	< 0.001
No, n (%)	682 (90.8)	1,397 (93.1)	699 (92.7)	669 (89.4)	612 (81.7)	599 (79.8)	570 (76.0)	554 (73.9)	
ABO blood group^b^									
A, n (%)	236 (37.8)	318 (28.7)	168 (27.5)	131 (21.7)	117 (20.7)	119 (21.7)	126 (23.7)	100 (20.1)	< 0.001
AB, n (%)	44 (7.0)	63 (5.7)	30 (4.9)	29 (4.8)	34 (6.0)	25 (4.6)	31 (5.8)	27 (5.4)	
B, n (%)	162 (25.9)	312 (28.1)	165 (27.0)	171 (28.4)	179 (31.6)	155 (28.3)	138 (25.9)	147 (29.6)	
O, n (%)	183 (29.3)	416 (37.5)	248 (40.6)	272 (45.1)	236 (41.7)	249 (45.4)	237 (44.5)	223 (44.9)	
Total energy intake (kcal/day), mean (± SD)	950.5 (± 174.7)	1171.3 (± 210)	1407.4 (± 219.9)	1581 (± 259.6)	1714 (± 233.3)	1870.3 (± 232.9)	2030.5 (± 254.4)	2345.7 (± 332.5)	
Tertile 1, n (%)	728 (96.9)	1,210 (80.7)	269 (35.7)	51 (6.8)	1 (0.1)	0 (0.0)	0 (0.0)	0 (0.0)	< 0.001
Tertile 2, n (%)	20 (2.7)	259 (17.3)	447 (59.3)	589 (78.7)	533 (71.2)	319 (42.5)	81 (10.8)	0 (0.0)	
Tertile 3, n (%)	3 (0.4)	31 (2.1)	38 (5.0)	108 (14.4)	215 (28.7)	432 (57.5)	669 (89.2)	750 (100.0)	

^a^n(column %), ^b^Based on available data, SD is standard deviation, BMI is body Mass Index (Asian category), min is minimum, max is maximum.

A U-shaped association between selenium intake and cancer risk was observed in the current analysis. A safe intake ranged from 110.8 to 124.4 µg/day (mean 117.8 µg/day). Compared to individuals with the safe intake of selenium, individuals with the lowest intake (i.e., 27.8–77.2 µg/day) were associated with an increased risk of cancer (OR = 3.78, 95% CI 2.89–4.95), *P*_*trend*_ < 0.001, and those with the highest intake (i.e., 169.1–331.7 µg/day) also had an increased cancer risk (OR = 1.86, 95% CI 1.45–2.39), *P*_*trend*_ = 0.003. We observed a similar U-shaped pattern in both men and women (Table 3).

**Table 3:** Association between selenium intake and risk of cancer overall and stratified by sex.

Selenium intake, µg/day, mean (min–max)	Overall			Men			Women		
	Case	Control	OR (95% CI)^a^	Case	Control	OR (95% CI)^a^	Case	Control	OR (95% CI)^a^
*P*_*trend*_^b^			< 0.001			0.002			0.022
67.4 (27.8–77.2) µg/day	599	152	3.78 (2.89, 4.95)	316	76	3.95 (2.73, 5.72)	283	76	3.10 (2.07, 4.66)
86.8 (77.3–95.0) µg/day	1063	437	2.41 (1.94, 3.00)	609	221	2.73 (2.03, 3.66)	454	216	1.98 (1.42, 2.76)
103.1 (95.1–110.7) µg/day	409	345	1.37 (1.10, 1.70)	235	182	1.42 (1.06, 1.89)	174	163	1.25 (0.90, 1.74)
117.8 (110.8–124.4) µg/day	324	424	1.00	191	226	1.00	133	198	1.00
130.9 (124.5–137.0) µg/day	314	435	1.01 (0.81, 1.25)	189	254	0.90 (0.68, 1.19)	125	181	1.23 (0.88, 1.72)
143.8 (137.1–151.0) µg/day	332	419	1.24 (0.99, 1.55)	202	257	1.04 (0.78, 1.40)	130	162	1.63 (1.15, 2.31)
159.2 (151.1–169.0) µg/day	345	405	1.50 (1.18, 1.91)	245	273	1.29 (0.95, 1.75)	100	132	1.91 (1.29, 2.85)
188.3 (169.1–331.7) µg/day	372	378	1.86 (1.45, 2.39)	280	268	1.58 (1.15, 2.16)	92	110	2.30 (1.50, 3.52)
*P*_*trend*_^c^			0.003			0.028			0.050
*P*_*heterogeneity*_						0.005			

Using an estimated mean intake of 117.8 µg/day (Range: 110.8–124.4 µg/day) as a Reference Group. Min is minimum; max is maximum, OR (95% CI): odds ratio (95% confidence interval).

^a^Model adjusted age groups (15–29, 30–39, 40–49, 50–59, 60–69, 70+), sex (if applicable), highest education level (primary, secondary, high school or higher), BMI (kg/m^2^, < 18.5, 18.5–< 23, 23–24.9, 25+), alcohol consumption (yes/no), family history of cancer (yes/no), smoking status (ever/never), history of diabetes (yes/no), coffee drinking (yes/no). and total energy intake (kcal/day, tertile), and four data collection periods.

^b^*P*_*trend*_ for estimates below mean intake (reference).

^c^*P*_*trend*_ for estimates above mean intake (reference).

In stratified analysis, a U-shaped pattern of association between selenium intake and cancer risk was stronger among participants with BMI < 23 kg/m^2^ and never smokers than BMI ≥ 23 kg/m^2^ and ever smokers (*P’s*_*heterogeneity*_ = 0.003 and 0.021, respectively). A U-shaped association between selenium intake and increased risk of cancer was observed in both never and ever-drinkers of alcohol (*P*_heterogeneity_ < 0.001) (Table 4).

**Table 4:** Association between selenium intake and risk of cancer, stratified by BMI, smoking status, and drinking status.

	*P*_*trend*_^b^	67.4 µg/day	86.8 µg/day	103.1 µg/day	117.8 µg/day	130.9 µg/day	143.8 µg/day	159.2 µg/day	188.3 µg/day	*P*_*trend*_^c^	*P*_*interaction*_
Range (min–max), µg/day		27.8–77.2 µg/day	77.3–95.0 µg/day	95.1–110.7 µg/day	110.8–124.4 µg/day	124.5–137.0 µg/day	137.1–151.0 µg/day	151.1–169.0 µg/day	169.1–331.7 µg/day		
By BMI status											
BMI < 23 kg/m^2^											
Case (n = 2945)		430	808	311	261	256	281	280	318		
Control (n = 2121)		97	303	232	303	313	315	297	261		
OR (95% CI)^a^	< 0.001	3.72 (2.71, 5.11)	2.44 (1.90, 3.14)	1.38 (1.08, 1.77)	1.00	1.03 (0.81, 1.30)	1.25 (0.97, 1.60)	1.48 (1.13, 1.95)	2.01 (1.51, 2.67)	< 0.001	
BMI ≥ 23 kg/m^2^											0.003
Case (n = 813)		169	255	98	63	58	51	65	54		
Control (n = 874)		55	134	113	121	122	104	108	117		
OR (95% CI)^a^	< 0.001	3.63 (2.16, 6.11)	2.45 (1.58, 3.80)	1.45 (0.95, 2.23)	1.00	1.12 (0.71, 1.76)	1.35 (0.83, 2.21)	1.76 (1.06, 2.92)	1.61 (0.94, 2.74)	0.146	
By smoking status											
Never smokers											
Case (n = 2190)		373	675	227	177	182	198	176	182		
Control (1822)		97	280	230	274	270	250	212	209		
OR (95% CI)^a^	< 0.001	3.72 (2.63, 5.27)	2.49 (1.88, 3.30)	1.26 (0.95, 1.67)	1.00	1.17 (0.89, 1.55)	1.59 (1.19, 2.13)	2.10 (1.52, 2.91)	2.49 (1.77, 3.49)	0.008	0.021
Ever smokers											
Case (n = 1568)		226	388	182	147	132	134	169	190		
Control (n = 1173)		55	157	115	150	165	169	193	169		
OR (95% CI)^a^	< 0.001	3.69 (2.39, 5.70)	2.27 (1.60, 3.22)	1.52 (1.08, 2.13)	1.00	0.79 (0.56, 1.11)	0.85 (0.59, 1.20)	0.95 (0.66, 1.37)	1.25 (0.86, 1.82)	0.089	
By alcohol drinking status											
Never drinker											
Case (n = 1969)		246	566	232	185	181	195	169	195		
Control (n = 1706)		96	277	226	255	247	212	195	198		
OR (95% CI)^a^	< 0.001	2.71 (1.91, 3.85)	2.13 (1.61, 2.83)	1.24 (0.94, 1.64)	1.00	1.07 (0.81, 1.42)	1.48 (1.10, 1.99)	1.64 (1.18, 2.27)	1.95 (1.39, 2.74)	0.028	< 0.001
Ever drinker											
Case (n = 1789)		353	497	177	139	133	137	176	177		
Control (n = 1289)		56	160	119	169	188	207	210	180		
OR (95% CI) ^a^	< 0.001	4.99 (3.23, 7.71)	2.76 (1.95, 3.92)	1.57 (1.12, 2.21)	1.00	0.93 (0.67, 1.29)	1.01 (0.71, 1.42)	1.39 (0.97, 2.00)	1.75 (1.20, 2.54)	0.020	

Using an estimated mean intake of 117.8 µg/day (Range: 110.8–124.4 µg/day) as a Reference Group. Min is minimum; max is maximum, OR (95% CI): odds ratio (95% confidence interval).

^a^ Model adjusted for age groups (15–29, 30–39, 40–49, 50–59, 60–69, 70+), sex (if applicable), highest education level (primary, secondary, high school or higher), BMI (kg/m^2^, < 18.5, 18.5–< 23, 23–24.9, 25+), alcohol consumption (yes/no), family history of cancer (yes/no), smoking status (ever/never), history of diabetes (yes/no), coffee drinking (yes/no). and total energy intake (kcal/day, tertile), and four data collection periods.

^b^*P*_*trend*_ for estimates below mean intake (Reference).

^c^*P*_*trend*_ for estimates above mean intake (Reference).

In an analysis of cancer-specific sites, a U-shaped association between selenium intake and cancer risk was seen in cancer sites of the stomach, colon, rectum, and lung cancers (Supplementary Table 1).

## Discussion

The new findings were that we found a U-shaped association between selenium intake and cancer risk. A safe intake ranged from 110.8 to 124.4 µg/day. A similar U-shaped association was also seen among men and women, ever and never-smokers, BMI < 23 kg/m^2^ and BMI ≥ 23 kg/m^2^, ever and never drinkers, cancer sites of the stomach, colon, rectum, and lung cancers.

We observed a safe intake of the micronutrient selenium, which protects against cancer, at a narrow range of 110.8–124.4 µg/day. People who eat less than this safe intake level increase their cancer risk gradually as their intake level decreases. People who eat higher than this level also have a continuous increase in cancer risk with increasing selenium intake, and the increase has the most robust statistical significance at the highest selenium intake from 169.1 to 331.7 µg/day. A U-shaped pattern of association between selenium intake and cancer risk was stronger among participants with BMI < 23 kg/m^2^ and never smokers than among those with BMI ≥ 23 kg/m^2^ and ever smokers would be explained by the differed sample size. The number of cancer patients was 2945 (BMI < 23 kg/m^2^) versus 813 (BMI ≥ 23 kg/m^2^) and 2190 (never smokers) versus 1586 (ever smokers).

The Recommended Dietary Allowance (RDA) for selenium is 55 μg/day for adults^1,15^. The other study also suggested that humans selenium intake levels of 60 μg/day to prevent type 2 diabetes^38^. This range from 55 to 60 μg/day is lower than a safe selenium intake in the present Vietnamese study population. The estimated lowest mean intake was 67.4 μg/day, ranging from 27.8 to 77.2 μg/day (The first quantile). Compared to individuals with the safe intake of selenium, individuals with the lowest mean intake were associated with an increased risk of cancer. A safe selenium intake might be associated with specific populations because the lowest quantile might be related to food deficiency of protein, amino acids, vitamins, minerals, and other essential micronutrients.

The present findings were consistent with previous studies. In a nested-case control study of 97 incident cancer cases and 184 controls in Poland, Narod et al.^36^ reported that individuals with serum selenium levels ≤ 70 µg/L (OR = 2.60, 95% CI 1.26, 5.35), 70.01–80 µg/L (OR = 1.13, 95% CI (0.59, 2.16), and > 90 µg/L (OR = 1.63, 95% CI 0.63, 4.19), compared to those with serum selenium level at 80.01–90 µg/L were suggestively increased risk of cancer; a similar U-shaped association to ours. Serum selenium concentration showed a U-shaped association with all-cause mortality, including cancer^37^.

A comprehensive review from the Cochrane Library on randomized clinical trials showed no protective effect of selenium against different types of cancers, including colorectal cancer, breast cancer, non‐melanoma skin cancer, bladder cancer, and lung cancer^24^. These findings might be limited because there was no data on a safe intake.

Different animal models have shown that selenium protects against cancer through various mechanisms, including effects on DNA stability, cell proliferation, necrosis, and apoptosis in both normal and malignant cells, as well as immune system and oxidative stress regulation^39,40^. However, other studies also reported that selenium might promote cancer cell transformation and progression^41–43^. For instance, in an experimental study by Chen et al.^43^ showed that vitamin E supplementation inhibited carcinogenesis through antioxidative properties. At the same time, a high dose of inorganic selenium might promote carcinogenesis by enhancing oxidative stress. This evidence of beneficial protection against cancer and cancer risk related to selenium intake might be explained by a U-sharp association in the present study. Prior studies showed that the primary sources of selenium are trace elements in crops, seafood, fish, animal products, or supplements^7,8^. Our study's primary sources of dietary selenium are rice and rice noodles, fish, pork, poultry, tofu, soy products, eggs, white radish, and cabbage (*data not shown*).

The current study has several limitations. Information bias and recall bias can be present in the case–control study design, mainly when participants had to place food they consumed 12 months before cancer diagnosis. However, our validation study of the FFQ showed that the Pearson correlation coefficients (*R*^2^) between the FFQ and 24-HDR ranged from 0.34 (for selenium) to 0.53 (for energy intake)^30,33^. Cancer patients might have changed their dietary habits during pre-clinical onset, leading to underestimating the association between selenium intake and cancer risk, an effect of latent disease in modifying metabolism (absorption, metabolism, and catabolism) of trace elements and their circulating levels, including selenium. A cautious interpretation of this association is thus warranted. Also, our findings might not be applied to the general population because cancer cases and control subjects were recruited in a hospital-based setting. Another concern is that our study exclusively focused on dietary selenium intake. However, previous studies found a moderate correlation between dietary intake of selenium, obtained from self-reported consumption of supplements, FFQ or nutritional records, and selenium levels in peripheral biomarkers, including blood, toenails, and hair^44–47^. Lastly, residual confounding might exist due to unmeasured confounding factors that could not be considered in the multivariable regression models. This study includes fewer controls than cases for the entire study population, which is another limitation of the study. However, by specific cancer sites of the stomach, colon, rectum, and lung cancers, the findings are consistent with the overall results.

Our study has several strengths. This study used data from a case–control design with a large sample size. From our household survey, we selected foods for FFQ that provided more than 90% of essential micronutrients, including selenium. All cancer cases were newly diagnosed patients with pathologic confirmed at the four leading tertiary hospitals in Hanoi, Vietnam. Also, a structured questionnaire allowed us to collect important information that could be used in the multivariable models to adjust for confounding factors.

In summary, a large case–control study in Vietnam found a U-shaped association between selenium intake and cancer risk. We observed a safe intake ranging from 110.8 to 124.4 µg/day. Further studies are warranted to replicate our findings in other populations to provide a comprehensive understanding of the role of selenium in cancer development.

## Supplementary Information

Supplementary Table 1.

## Data Availability

The datasets used and analyzed during the current study are available from the corresponding author upon reasonable request.
