# Supplementary material for: A U-shaped association between selenium intake and cancer risk
Source: Sci Rep. 2024 Sep 13;14:21378. doi: 10.1038/s41598-024-66553-5 (PMC11399399; doi:10.1038/s41598-024-66553-5)
Supplement: Supplementary file 1 — Supplementary Table 1. [file 41598_2024_66553_MOESM1_ESM.docx]

**A U-shaped Association between Selenium Intake and Cancer Risk**

**Supplementary Table 1. Association Between Selenium Intake and Risk of** **Specific Cancer Site**

|  | *P_trend_ ^b^* | 67.4 µg/day | 86.8 µg/day | 103.1 µg/day | 117.8 µg/day | 130.9 µg/day | 143.8 µg/day | 159.2 µg/day | 188.3 µg/day | *P_trend_ ^c^* |
| --- | --- | --- | --- | --- | --- | --- | --- | --- | --- | --- |
| Range (min-max), µg/day |  | 27.8-77.2 µg/day | 77.3-95.0 µg/day | 95.1-110.7 µg/day | 110.8-124.4 µg/day | 124.5-137.0 µg/day | 137.1-151.0 µg/day | 151.1-169.0 µg/day | 169.1-331.7 µg/day |  |
| Stomach Cancer | | |  |  |  |  |  |  |  |  |
| Case (n=1182) |  | 95 | 181 | 125 | 119 | 152 | 158 | 185 | 167 |  |
| Control (n=2995) |  | 152 | 437 | 345 | 424 | 435 | 419 | 405 | 378 |  |
| OR (95% CI) ^a^ | 0.017 | 1.84 (1.25, 2.72) | 1.46 (1.06, 2.00) | 1.33 (0.97, 1.82) | 1.00 | 1.03 (0.77, 1.38) | 1.24 (0.91, 1.69) | 1.58 (1.13, 2.19) | 1.60 (1.12, 2.27) | 0.078 |
| Colon Cancer |  |  |  |  |  |  |  |  |  |  |
| Case (n=567) |  | 67 | 112 | 73 | 62 | 56 | 70 | 58 | 69 |  |
| Control (n=2995) |  | 152 | 437 | 345 | 424 | 435 | 419 | 405 | 378 |  |
| OR (95% CI) ^a^ | 0.003 | 2.12 (1.35, 3.33) | 1.38 (0.94, 2.02) | 1.23 (0.84, 1.80) | 1.00 | 0.99 (0.66, 1.47) | 1.5 (1.01, 2.24) | 1.46 (0.94, 2.28) | 2.08 (1.32, 3.28) | 0.002 |
| Rectal Cancer |  |  |  |  |  |  |  |  |  |  |
| Case (n=482) |  | 46 | 93 | 40 | 51 | 52 | 61 | 66 | 73 |  |
| Control (n=2995) |  | 152 | 437 | 345 | 424 | 435 | 419 | 405 | 378 |  |
| OR (95% CI) ^a^ | 0.001 | 2.19 (1.32, 3.65) | 1.79 (1.18, 2.72) | 0.96 (0.61, 1.51) | 1.00 | 0.95 (0.62, 1.44) | 1.21 (0.79, 1.86) | 1.45 (0.92, 2.29) | 1.78 (1.11, 2.86) | 0.145 |
| Lung Cancer |  |  |  |  |  |  |  |  |  |  |
| Case (n=225) |  | 62 | 117 | 20 | 7 | 4 | 4 | 4 | 7 |  |
| Control (n=2995) |  | 152 | 437 | 345 | 424 | 435 | 419 | 405 | 378 |  |
| OR (95% CI) ^a^ | <0.001 | 11.05 (4.4, 27.77) | 6.84 (2.91, 16.08) | 1.81 (0.72, 4.53) | 1.00 | 1.21 (0.34, 4.31) | 2.11 (0.58, 7.73) | 2.83 (0.75, 10.77) | 6.11 (1.86, 20.06) | 0.281 |
| Other cancers |  |  |  |  |  |  |  |  |  |  |
| Case (n=971) |  | 159 | 271 | 75 | 52 | 20 | 26 | 19 | 41 |  |
| Control (n=2995) |  | 152 | 437 | 345 | 424 | 435 | 419 | 405 | 378 |  |
| OR (95% CI) ^a^ | <0.001 | 9.74 (5.80, 16.35) | 3.88 (2.48, 6.08) | 1.68 (1.08, 2.62) | 1.19 (0.76, 1.85) | 1.00 | 1.11 (0.67, 1.85) | 1.10 (0.63, 1.90) | 1.88 (1.14, 3.10) | 0.078 |

Using an estimated mean intake of 117.8 µg/day (Range: 110.8-124.4 µg/day) as a Reference Group. Min is minimum; max is maximum, OR (95% CI): odds ratio (95% confidence interval)

^a^ Model adjusted for age groups (15-29, 30-39, 40-49, 50-59, 60-69, 70+), sex (if applicable), highest education level (primary, secondary, high school or higher), BMI (kg/m^2^, <18.5, 18.5-<23, 23-24.9, 25+), alcohol consumption (yes/no), family history of cancer (yes/no), smoking status (ever/never), history of diabetes (yes/no), coffee drinking (yes/no). and total energy intake (kcal/day, tertile), and data collection periods

^b^ *P_trend_* for estimates below mean intake (Reference); ^c^ *P_trend_* for estimates above mean intake (Reference).
